# Glutaminase 2 negatively regulates the PI3K/AKT signaling and shows tumor suppression activity in human hepatocellular carcinoma

**DOI:** 10.18632/oncotarget.1862

**Published:** 2014-03-26

**Authors:** Juan Liu, Cen Zhang, Meihua Lin, Wei Zhu, Yingjian Liang, Xuehui Hong, Yuhan Zhao, Ken H. Young, Wenwei Hu, Zhaohui Feng

**Affiliations:** ^1^ Department of Radiation Oncology; ^2^ Department of Pediatrics, Rutgers Cancer Institute of New Jersey, Rutgers University, New Brunswick, NJ, USA; ^3^ Department of Hematopathology, University of Texas MD Anderson Cancer Center, Houston, TX, USA; ^4^ These two authors contributed equally to this work

**Keywords:** p53, GLS2, tumor suppression, PI3K/AKT, Hepatocellular carcinoma

## Abstract

The tumor suppressor p53 and its signaling pathway play a critical role in tumor prevention. As a direct p53 target gene, the role of glutaminase 2 (GLS2) in tumorigenesis is unclear. In this study, we found that GLS2 expression is significantly decreased in majority of human hepatocellular carcinoma (HCC). Restoration of GLS2 expression in HCC cells inhibits the anchorage-independent growth of cells and reduces the growth of HCC xenograft tumors. Interestingly, we found that GLS2 negatively regulates the PI3K/AKT signaling, which is frequently activated in HCC. Blocking the PI3K/AKT signaling in HCC cells largely abolishes the inhibitory effect of GLS2 on the anchorage-independent cell growth and xenograft tumor growth. The *GLS2* promoter is hypermethylated in majority of HCC samples. CpG methylation of *GLS2* promoter inhibits *GLS2* transcription, whereas reducing the methylation of GLS2 promoter induces GLS2 expression. Taken together, our results demonstrate that GLS2 plays an important role in tumor suppression in HCC, and the negative regulation of PI3K/AKT signaling contributes greatly to this function of GLS2. Furthermore, hypermethylation of *GLS2* promoter is an important mechanism contributing to the decreased GLS2 expression in HCC.

## INTRODUCTION

Tumor suppressor p53 plays a crucial role in tumor prevention, including hepatocellular carcinoma (HCC) [1-3]. p53 is the most frequently-mutated gene in human tumors; over 50% of human tumors harbor mutations in the p53 gene. In HCC, around 40-60% of HCCs contain DNA mutations in the p53 gene [4, 5]. Disruption of normal *p53* function is in many circumstances a prerequisite for the development or progression of tumors [6, 7]. As a transcription factor, p53 mainly exerts its tumor suppression function through its transcriptional regulation of its downstream target genes. Through the regulation of the expression of many downstream target genes, p53 regulates cell cycle arrest, apoptosis, senescence, cellular energy metabolism and anti-oxidant defense, all of which contribute to the role of p53 in tumor suppression [1-3, 8].

Glutaminase 2 (GLS2) is a liver-type mitochondrial glutaminase, which can catalyze the hydrolysis of glutamine to glutamate in mitochondria in cells [9, 10]. GLS2 is specifically expressed in very few tissues, including the liver. To date, little is known about the biological functions of GLS2 in cells except for its function as a glutaminase. Recently, *GLS2* was identified as a novel p53 downstream target gene by our group and another group [11, 12]. p53 induces the GLS2 expression under both stressed and non-stressed conditions. Importantly, GLS2 mediates the functions of p53 in regulation of energy metabolism and anti-oxidant defense [11, 12]. Considering the critical role of p53 and its pathway in tumor suppression, as a novel p53 target gene, GLS2 might play an important role in tumor suppression. However, the role of GLS2 in tumorigenesis is not well-understood.

HCC is the fifth most frequent cancer worldwide [13-15]. HCC is a highly malignant tumor type with average survival rates less than 1 year following diagnosis. One main reason for the high HCC mortality is because most patients are diagnosed when the disease is already at an advanced stage, and the cancerous tissue cannot be surgically removed [13-15]. Therefore, further understanding the molecular mechanism of liver tumorignesis will provide potential molecular biomarkers for early diagnosis and novel therapeutic strategies for HCC.

In this study, we investigated the role of GLS2 in liver tumorigenesis. Our results demonstrated that GLS2 protein levels were significantly decreased in majority of HCCs that we examined. GLS2 inhibited the anchorage-independent growth of HCC cells and the growth of HCC xenograft tumors. Furthermore, GLS2 negatively regulated the PI3K/AKT signaling, which is frequently activated in various tumors, including HCC, and plays a pivotal role in tumorigenesis [16-18]. Blocking the PI3K/AKT signaling largely abolished the inhibitory effect of GLS2 liver tumorigenesis. CpG hypermethylation in gene promoters is an important epigenetic mechanism that contributes to decreased expression of tumor suppressor genes in cancer, including HCC [19-21]. Our results strongly suggested that hypermethylation of *GLS2* promoter is an important mechanism that contributes to the down-regulation of GLS2 expression in HCC. Taken together, results from this study demonstrated an important role of GLS2 in tumor suppression in HCC through its negative regulation of the PI3K/AKT signaling.

## RESULTS

### The GLS2 protein expression is frequently decreased in human primary HCC

Liver is one of few tissues that specifically express GLS2. Previously, we examined the levels of GLS2 mRNA in a set of primary HCCs at different stages, and found that GLS2 mRNA levels were greatly decreased in majority of primary HCCs compared with normal liver tissues or tumor adjacent liver tissues [11]. These results are consistent with the results from another study [12]. However, it was unclear whether the change of GLS2 protein levels is consistent with the change of GLS2 mRNA levels in HCCs.

**Figure 1:**
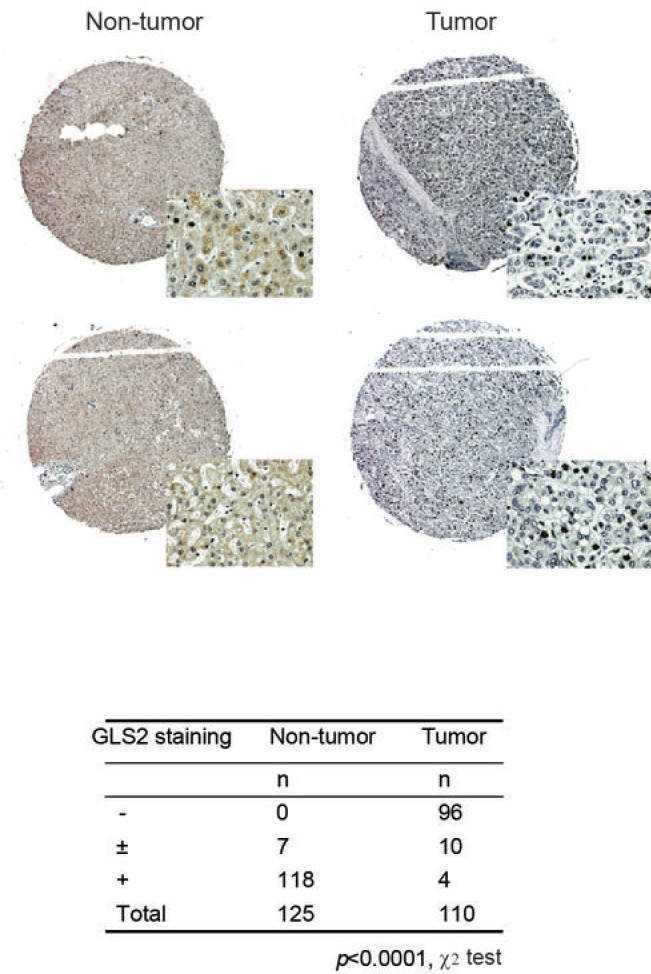
The decreased protein expression of GLS2 in human primary HCCs The GLS2 protein expression in HCC samples in three tissue microarrays (TMAs; US Biomax) as measured by IHC assays. The three TMAs contain totally 110 primary HCCs and 125 non-tumor liver samples. Upper panels: Representative IHC staining of GLS2 in 2 HCCs which showed negative staining (-) and 2 non-tumor liver tissues which showed positive staining (+). Lower panel: IHC staining results in the TMA. -: 0% positive staining cells; ±: <10% positive staining cells; +: ≥10% positive staining cells. The *p* values were calculated using χ2 tests. The clinico-pathological information of HCC samples was presented in Supplementary Tables S1 & S2.

To investigate whether GLS2 protein expression is decreased in HCCs, we analyzed the levels of GLS2 protein in two different sets of primary HCC samples by immunohistochemistry (IHC) staining assays. One set of samples was provided by US Biomax (Rockville, MD), which includes totally 110 primary HCCs and 125 non-tumor liver tissues in three tissue microarrays (TMAs). Another set of samples was collected at University of Texas MD Anderson Cancer Center, which includes 21 pairs of primary HCCs and their matched adjacent non-tumor liver tissues. As shown in Figure 1, IHC staining results showed that in 125 non-tumor liver samples in the TMA, 118 samples showed positive staining for GLS2 (+; ≥10% positive staining cells) and 7 samples showed weak staining for GLS2 (±; <10% positive staining cells). In contrast, in 110 HCC samples in the TMA, 96 HCC samples showed negative for GLS2 (-; 0% positive staining cells), and 10 samples showed weak staining for GLS2 (±), whereas only 4 HCC samples showed positive staining for GLS2 (+) (*p*<0.0001; HCCs vs. non-tumor liver tissues). The decreased expression of GLS2 protein appeared to be a common event in HCCs, which was observed in majority of HCCs at different stages and different histological grades (Supplementary Tables S1 and S2).

Similar results were observed in another set of HCC samples. IHC staining results showed that in 21 primary HCC samples, 19 samples showed negative staining for GLS2 (-), 1 showed weak staining (±) and 1 showed positive staining (+). In contrast, in the matched 21 adjacent non-tumor liver tissues, 20 samples showed positive staining for GLS2 (+) and 1 showed weak staining for GLS2 (±) (Supplementary Figure S1A; *p*<0.0001; HCCs vs. adjacent non-tumor liver tissues). Again, the decreased expression of GLS2 protein was observed in majority of HCCs at different stages and different histological grades (Supplementary Table S3). Consistent with the results from IHC staining, results from Taqman real-time PCR assays showed that GLS2 mRNA levels were clearly decreased in majority of HCCs; 18 of 21 HCCs showed decreased mRNA levels compared with their matched adjacent non-tumor liver tissues (the cut-off is 2-fold difference). The average reduction fold was ~8-fold (Supplementary Figure S1B). Taken together, these results clearly demonstrated that GLS2 expression is significantly decreased at both mRNA and protein levels in majority of primary HCCs, which strongly suggests a potential important role of GLS2 in tumor suppression of HCC.

### GLS2 inhibits anchorage-independent growth of HCC cells and the growth of HCC xenograft tumors

**Figure 2:**
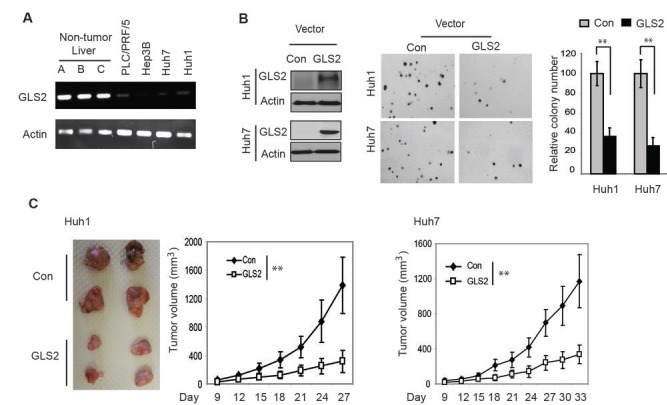
GLS2 inhibits anchorage-independent growth of HCC cells and growth of HCC xenograft tumors (A). GLS2 mRNA expression in different HCC cell lines and 3 non-tumor liver tissues (provided by Origene) as measured by semi-quantitative RT-PCR. (B) Ectopic expression of GLS2 in Huh1 and Huh7 cells inhibited anchorage-independent growth of cells in soft agar. Left panel: Huh1 and Huh7 cells were stably transduced with pLPCX-GLS2 vectors expressing GLS2 or control empty vectors. GLS2 expression in cells was detected by Western-blot assays. Middle panel: Representative images of anchorage-independent growth of cells in soft agar. Right Panel: relative colony number of cells with ectopic GLS2 expression or control cells in soft agar. Data are presented as mean ± SD (n = 5). **: *p*<0.001; Student *t*-tests. (C) Ectopic expression of GLS2 in Huh1 and Huh7 cells inhibited the growth of xenograft tumors. Left panel: Representative images of xenograft tumors formed by Huh1 cells with or without ectopic GLS2 expression at day 27 after inoculation of cells. Middle panel: The growth curves of xenograft tumors formed by Huh1 cells with or without ectopic GLS2 expression. Right panel: The growth curves of xenograft tumors in nude mice formed by Huh7 cells with or without ectopic GLS2 expression. Data are presented as mean ± SD (n=10); **: *p*<0.001; ANOVA followed by Student’s *t*-tests.

We further investigated whether GLS2 plays a role in tumor suppression in HCC. As shown in Figure 2A, compared with non-tumor liver tissues (3 tissues from Origene), GLS2 mRNA levels were clearly decreased in different HCC cell lines, including Huh1, Huh7, PLC/PRF/5, and Hep3B cells. To investigate the role of GLS2 in HCC, GLS2 was ectopically expressed in Huh1 and Huh7 cells by stable transduction of a retroviral vector, which expresses GLS2 (Figure 2B, left panel). Control cells were transduced with empty vectors. These cell lines were then employed for both anchorage-independent growth assays in soft agar and xenograft tumor assays in nude mice. As shown in Figure 2B (middle and right panels), ectopic expression of GLS2 in Huh1 and Huh7 cells significantly inhibited the anchorage-independent growth of cells in soft agar compared with their control cells. Similarly, ectopic expression of GLS2 greatly reduced the growth of xenograft tumors formed by Huh1 and Huh7 cells compared with tumors formed by their control cells (Figure 2C).

**Figure 3:**
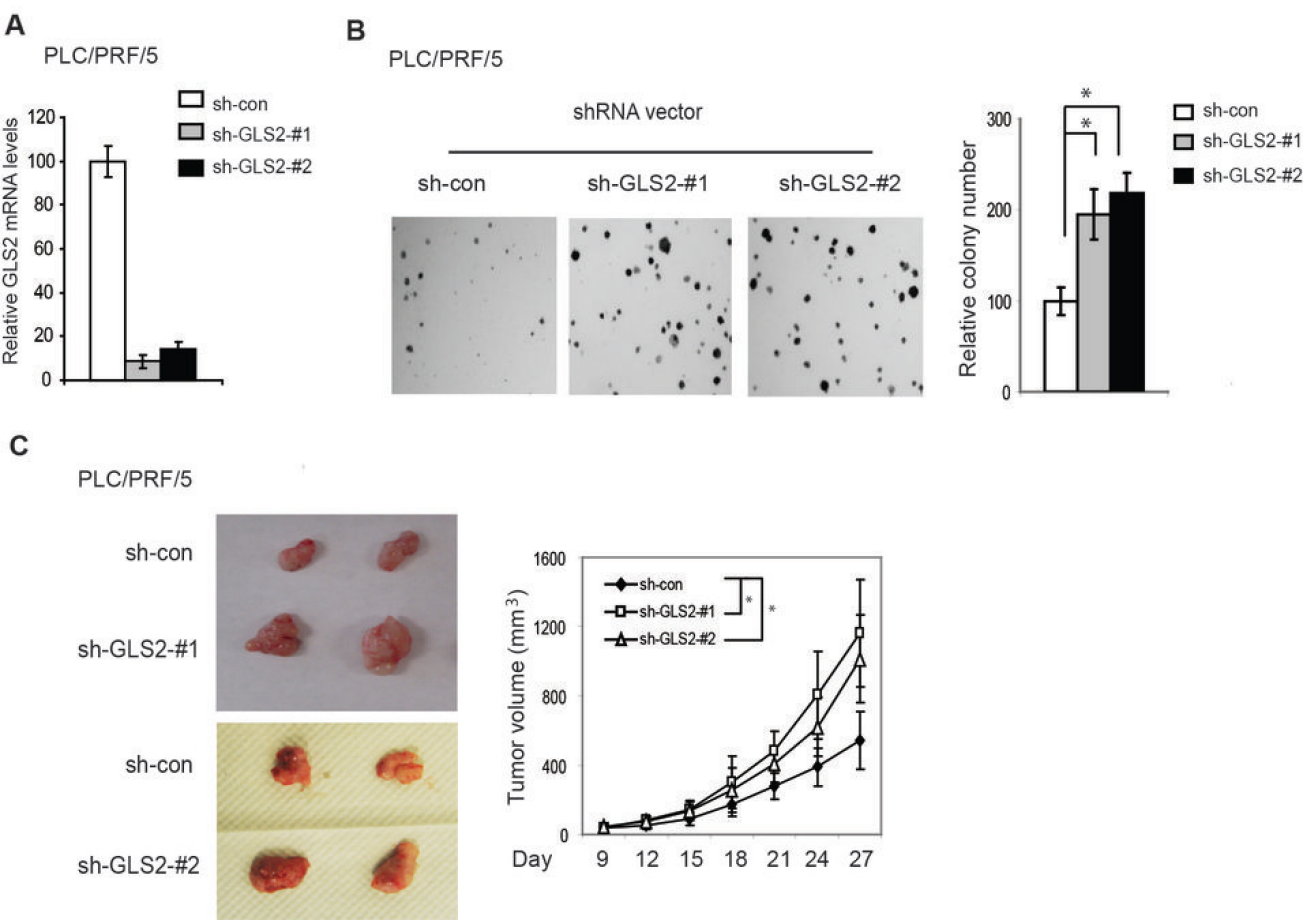
Knockdown of GLS2 promotes anchorage-independent growth of HCC cells and growth of HCC xenograft tumors (A) Knockdown of endogenous GLS2 in PLC/PRF/5 cells measured by Taqman real-time PCR assays. Cells were stably transduced with control shRNA vectors (sh-con) or 2 different vectors against GLS2 (sh-GLS2-#1 and sh-GLS2-#2). The mRNA levels of GLS2 were measured by real-time PCR and normalized with Actin. The relative levels of GLS2 in control cells were designated as 1. (B) Knockdown of GLS2 promoted anchorage-independent growth of PLC/PRF/5 cells in soft agar. Left panel: Representative images of anchorage-independent growth of cells in soft agar. Right panel: relative colony number of cells with GLS2 knockdown or control cells in soft agar. (C) Knockdown of GLS2 in PLC/PRF/5 cells promoted the growth of xenograft tumors. Left panel: Representative images of xenograft tumors formed by control cells or cells with stable GLS2 knockdown by 2 different shRNA vectors at day 27 after inoculation of cells. Right Panel: The growth curves of xenograft tumors formed by PLC/PRF/5 cells with or without GLS2 knockdown. Data are presented as mean ± SD (n = 3 in A; n=5 in B; n=10 in C). * *p*<0.01. Student’s *t*-tests in B; ANOVA followed by Student’s *t*-tests in C.

To investigate whether loss of GLS2 expression promotes tumorigenesis in HCC, endogenous GLS2 in PLC/PRF/5 cells was knocked down by stable transduction of shRNA vectors against GLS2 (Figure 3A). PLC/PRF/5 cell line was used for shRNA knockdown since it shows relatively higher mRNA levels of GLS2 compared with other HCC cell lines that we tested (Figure 2A). To avoid off-target effects, two different shRNA vectors against GLS2 were employed (Figure 3A). As shown in Figure 3B, knockdown of GLS2 in PLC/PRF/5 cells significantly promoted the anchorage-independent cell growth in soft agar compared with their control cells transduced with control shRNA. Furthermore, knockdown of GLS2 also clearly promoted the growth of xenograft tumors formed by PLC/PRF/5 cells (Figure 3C). These results together strongly suggest an important role of GLS2 in tumor suppression in HCC.

### GLS2 negatively regulates the PI3K/AKT signaling

**Figure 4:**
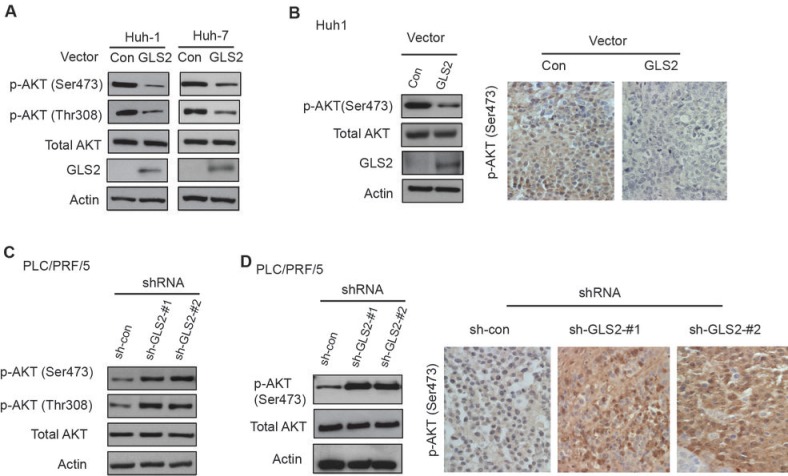
GLS2 negatively regulates the PI3K/AKT signaling in HCC cells (A) Ectopic expression of GLS2 reduced AKT phosphorylation at Ser473 and Thr308 in Huh1 and Huh7 cells as measured by Western-blot assays. (B) Ectopic expression of GLS2 reduced AKT phosphorylation at Ser473 in xenograft tumors formed by Huh1 cells as detected by Western-blot (left) and IHC staining (right) assays, respectively. (C) GLS2 knockdown by 2 different shRNA vectors increased AKT phosphorylation at Ser473 and Thr308 in PLC/PRF/5 cells as measured by Western-blot assays. GLS2 knockdown was presented in Figure 3A. (D) GLS2 knockdown by shRNA vectors increased AKT phosphorylation at Ser473 in xenograft tumors formed by PLC/PRF/5 cells as detected by Western-blot (left) and IHC staining (right) assays, respectively.

The PI3K/AKT signaling is frequently activated in various tumors, including HCC, which plays a critical role in promoting tumor cell growth, proliferation and survival [16, 17]. The PI3K/AKT signaling was reported to be activated in over 50% of HCCs [18]. Interestingly, we found that GLS2 negatively regulated the PI3K/AKT signaling in HCC cells. It is well-established that phosphorylation of AKT at Ser473 and Thr308 leads to the activation of AKT [16, 17]. Ectopic GLS2 expression in Huh1 and Huh7 cells clearly reduced the phosphorylation of AKT at Ser473 and Thr308 (Figure 4A). The down-regulation of AKT activities by GLS2 was also observed in HCC xenograft tumors; results from both Western-blot and IHC staining assays showed that AKT phosphorylation at Ser473 was clearly reduced in xenograft tumors formed by Huh1 cells with ectopic expression of GLS2 compared with tumors formed by control cells (Figure 4B). Furthermore, knockdown of the endogenous GLS2 in PLC/PRF/5 cells clearly enhanced the phsphorylation of AKT at Ser473 and Thr308 (Figure 4C). Consistently, AKT phosphorylation at Ser473 was clearly increased in xenograft tumors formed by PLC/PRF/5 cells with GLS2 knockdown compared with tumors formed by control cells (Figure 4D). Considering the critical role of the oncogenic PI3K/AKT signaling in HCC, the negative regulation of the PI3K/AKT signaling by GLS2 could contribute greatly to the tumor suppression activity of GLS2 in HCC.

### The negative regulation of the PI3K/AKT signaling by GLS2 contributes to tumor suppression activity of GLS2 in HCC

**Figure 5:**
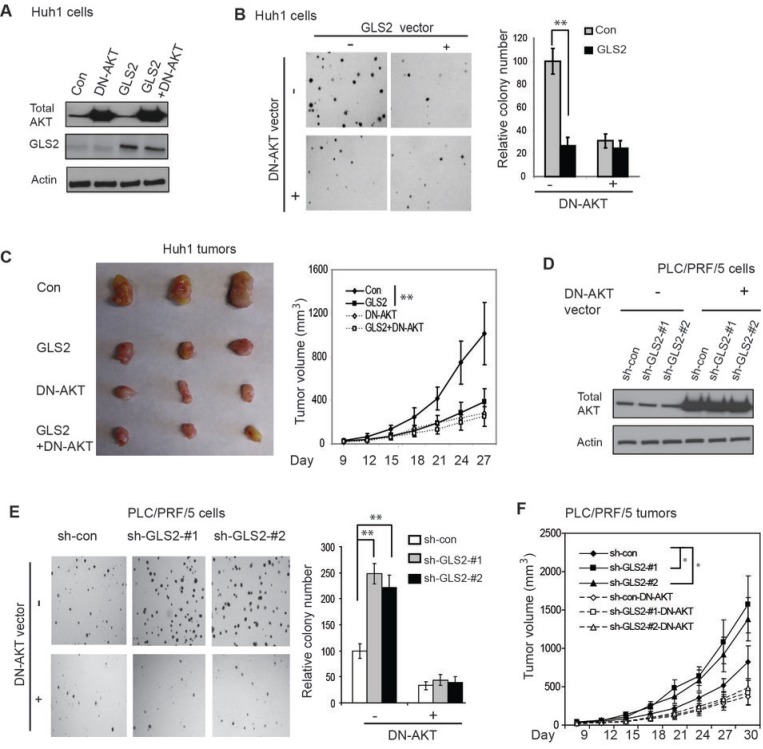
GLS2 negatively regulates the PI3K/AKT signaling to inhibit anchorage-independent growth of HCC cells and growth of xenograft HCC tumors (A) Ectopic expression of GLS2 and/or a dominant negative AKT (DN-AKT, K179M) in Huh1 cells measured by Western-blot assays. Cells were stably transduced with pLPCX-GLS2 vectors and/or pLHCX-DN-AKT (K179M) vectors. Control cells were transduced with control empty vectors (Con). (B) Expression of DN-AKT largely abolished the inhibitory effect of GLS2 on anchorage-independent growth of Huh1 cells in soft agar. Left panels: Representative images of anchorage-independent growth of Huh1 cells in soft agar. Right Panel: Relative colony number of Huh1 cells in soft agar. (C) DN-AKT largely abolished the inhibitory effect of GLS2 on the growth of xenograft tumors formed by Huh1 cells. Left panels: Representative images of xenograft tumors formed by Huh1 cells at day 27 after inoculation of cells. Right Panel: The growth curves of xenograft tumors formed by Huh1 cells. (D) Ectopic expression of the DN-AKT (K179M) in PLC/PRF/5 cells with GLS2 knockdown measured by Western-blot assays. PLC/PRF/5 cells with GLS2 knockdown (shown in Figure 3A) were transduced with the DN-AKT vectors. (E & F). DN-AKT largely abolished the promoting effect of GLS2 knockdown on anchorage-independent growth of PLC/PRF/5 cells (E) and on the growth of xenograft tumors formed by PLC/PRF/5 cells (F). Left panels in E: Representative images of anchorage-independent growth of PLC/PRF/5 cells in soft agar. Right panel in E: Relative colony number of PLC/PRF/5 cells. Data are presented as mean ± SD (n = 5 in B & E; n=10 in C & F). **: *p*<0.001; *: *p*<0.01; Student’s *t*-tests in B & E, and ANOVA followed by Student’s *t*-tests in C & F.

We further investigated whether negative regulation of the PI3K/AKT signaling by GLS2 contributes to GLS2’s role in tumor suppression in HCC. To this end, Huh1 cells with ectopic GLS2 expression and control cells were stably transduced with a vector expressing a dominant negative AKT (DN-AKT; K179M), which inhibits AKT activities in cells [22, 23] (Figure 5A). The DN-AKT expression clearly inhibited the anchorage-independent growth of Huh1 cells (Figure 5B) and the growth of Huh1 xenograft tumors (Figure 5C). Notably, DN-AKT expression largely abolished the inhibitory effect of GLS2 on the anchorage-independent growth and the growth of xenograft tumors. The differences in colony number and growth rates of tumors between cells co-expressing GLS2 and DN-AKT (GLS2+DN-AKT) and cells expressing DN-AKT (DN-AKT) were much less pronounced compared with the differences between cells expressing GLS2 (GLS2) and control cells (Con) (Figure 5B&C).

To investigate whether blocking the PI3K/AKT signaling can largely abolish the promoting effect of GLS2 knockdown on the anchorage-independent growth of HCC cells and the growth of HCC xenograft tumors, the DN-AKT (K179M) was stably expressed in PLC/PRF/5 cells with stable GLS2 knockdown (Figure 5D). Whereas GLS2 knockdown in PLC/PRF/5 cells clearly increased the colony number in soft agar and the growth rates of xenograft tumors, DN-AKT expression largely abolished the promoting effect of GLS2 knockdown on the anchorage-independent growth (Figure 5E) and the growth of xenograft tumors (Figure 5F). These results strongly suggest that the negative regulation of PI3K/AKT signaling by GLS2 contributes greatly to the tumor suppressive activity of GLS2 in HCC.

### Hypermethylation of the CpG sites in the promoter region of *GLS2* in human HCC cells and primary HCCs

**Figure 6:**
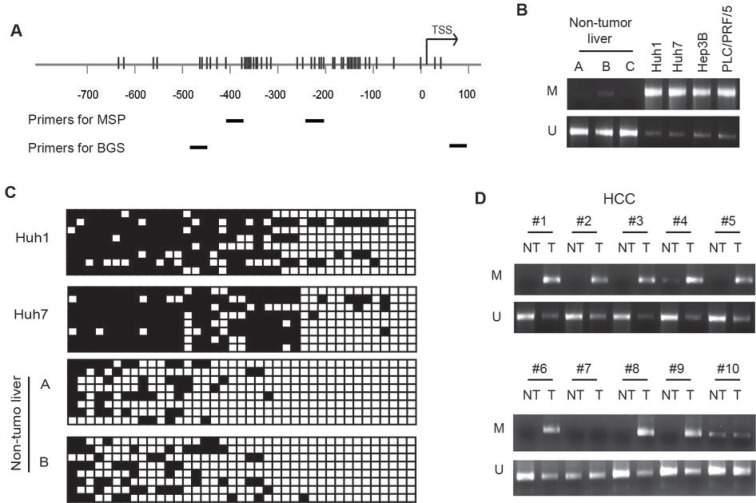
Hypermethylation of the GLS2 promoter in HCC cells and primary HCCs (A) CpG sites in the *GLS2* promoter region. CpG sites and their genomic positions in the *GLS2* promoter region are represented by vertical lines. Nucleotide positions are numbered relative to the transcriptional start site (TSS; +1). Positions of primers for methylation-specific PCR (MSP) and bisulfite genomic sequencing (BGS) assays are labeled. (B) Hypermethylation of the *GLS2* promoter in HCC cells detected by MSP analysis. The methylation status of *GLS2* promoter in 4 HCC cell lines and 3 non-tumor liver tissues (provided by Origene) were analyzed by MSP. U: PCR with unmethylation-specific primers; M: PCR with methylation-specific primers. (C) Hypermethylation of the *GLS2* promoter in HCC cell lines analyzed by BGS analysis. Eight clones of PCR products of bisulfite-treated DNA from 2 HCC cell lines and 2 non-tumor liver tissues (Origene) were sequenced. Black and white squares represent methylation and unmethylation, respectively. (D) Hypermethylation of the *GLS2* promoter in primary HCCs detected by MSP. The 21 pairs of primary HCCs and their matched non-tumor liver tissues were analyzed by MSP. Representative images are MSP results of samples #1-10. NT: Non-tumor liver tissue; T: Tumor.

Hypermethylation of the promoter is an important epigenetic mechanism that contributes to the decreased expression of tumor suppressor genes in human cancer, including HCC [19-21]. The promoter region of *GLS2* gene is a CpG rich domain (Figure 6A). To investigate whether promoter hypermethylation is an important mechanism contributing to the reduced expression of GLS2 in HCC, the methylation status of *GLS2* promoter was analyzed in human HCC cell lines and primary HCCs. First, methylation-specific PCR (MSP) assays were employed to analyze the methylation status of the *GLS2* promoter in multiple HCC cell lines, including Huh1, Huh7, Hep3B and PLC/PRF/5. Compared with the non-tumor liver tissues (3 tissues from Origene), all of these cell lines showed clearly decreased mRNA levels of GLS2 (Figure 2A). MSP analysis revealed that methylation was detected in the *GLS2* promoter region in all of these HCC cell lines. In contrast, no clear methylation or much lower methylation in *GLS2* promoter region was detected in these 3 non-tumor liver tissues (Figure 6B). To examine the methylation status of the *GLS2* promoter in more detail, bisulfite genomic sequencing (BGS) assays were used to analyze the 2 non-tumor liver samples (Origene), Huh1 and Huh7 cells (Figure 6C). Consistent with MSP results, BGS analysis showed that CpG sites in the GLS2 promoter region were extensively methylated on majority of CpG sites that we analyzed in these 2 HCC cell lines. In contrast, for these 2 non-tumor liver tissues, no methylation or much lower levels of methylation were observed in these CpG sites that showed hypermethylation in those HCC cells (Figure 6C).

We further investigated whether the *GLS2* promoter is hypermethylated in primary HCCs. MSP assays were employed to detect the methylation levels in *GLS2* promoter in the above-mentioned 21 pairs of HCCs and their adjacent non-tumor liver tissues. MSP assays showed that the *GLS2* promoter was hypermethylated in majority of HCCs. Compared with their matched non-tumor tissues, much higher levels of methylation (>2 fold higher) were detected in 16 of 21 tumors, similar levels of methylation were detected in 2 tumors (e.g. #10) and no clear methylation (e.g. # 7) was detected in 3 tumors (Figure 6D). The hypermethylation of these 2 non-tumor liver tissues could be caused by the contamination of tumor cells in non-tumor tissues. Furthermore, *GLS2* promoter hypermethylation is significantly associated with decreased GLS2 expression in HCCs (*p*<0.001; χ^2^ test). These results strongly suggest that hypermethylation of *GLS2* promoter is an important mechanism contributing to the decreased expression of GLS2 in HCC.

### CpG methylation inhibits the transcriptional activity of the GLS2 promoter

**Figure 7:**
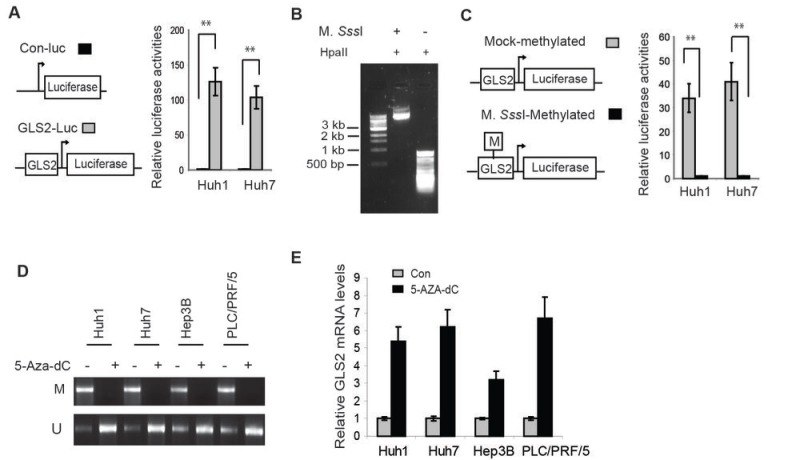
Promoter hypermethylation reduces GLS2 expression in HCC cells (A) *GLS2* promoter activated the luciferase reporter vectors in Huh1 and Huh7 cells. Left panel: Schematic representations of control luciferase reporter vectors (Con-Luc) and reporter vectors containing *GLS2* promoter region (GLS2-Luc). The relative luciferase activities of control reporter were designated as 1. (B) Complete methylation of the pGL2-GLS2 reporter vectors as revealed by digestion of HpaII. The vectors methylated by M. *Sss*I or mock-methylated were digested with HpaII and separated on an agrose gel. (C) Methylation of *GLS2* promoter reduced the luciferase activities of reporter vectors in Huh1 and Huh7 cells. Left panel: Schematic representations of the unmethylated pGL2 luciferase reporter vectors containing methylated or mock-methylated *GLS2* promoter. The relative luciferase activities of the reporter vectors containing methylated *GLS2* promoter were designated as 1. (D) 5-Aza-dC reduced methylation of *GLS2* promoter in HCC cells as measured by MSP assays. Cells were treated with 5-Aza-dC (5 μM) or DMSO for 7 days. U: PCR with unmethylation-specific primers; M: PCR with methylation-specific primers. (E) 5-Aza-dC induced GLS2 mRNA levels in HCC cells. GLS2 mRNA levels were measured by Taqman real-time PCR assays and normalized with Actin. The relative GLS2 levels in control cells treated with DMSO were designated as 1. Data are presented as mean ± SD (n = 3). **: *p*<0.001; Student *t*-tests.

To directly investigate the effect of CpG hypermethylation on transcriptional activity of *GLS2* promoter, *GLS2* promoter region (-458 to +86 relative to the transcription start site) was amplified by PCR and subcloned into the promoter region of pGL2 luciferase reporter vectors. Luciferase reporter assays showed that the insertion of *GLS2* promoter clearly activated the luciferase activities of the reporter vectors by over 100-fold in Huh1 and Huh7 cells compared with control reporter vectors without insertion of *GLS2* promoter (Figure 7A). The reporter vectors were treated with CpG methyltransferase M. SssI to methylate cytosine residues of CpG in the vectors [24, 25]. As a control, the vectors were mock-methylated. The completion of CpG methylation was confirmed by digestion of HpaII, a methylation-sensitive restriction enzyme (Figure 7B). The *GLS2* promoter DNA fragments cut from methylated and mock-methylated PGL2 vectors were then re-inserted into unmethylated pGL2 vectors by ligation reactions. After ligation reactions, the unmethylated pGL2 vectors containing methylated or mock-methylated *GLS2* promoter were transfected into HCC cells for luciferase reporter assays. Reporter vectors containing methylated *GLS2* promoter showed much weaker luciferase activities compared with vectors containing mock-methylated *GLS2* promoter; *GLS2* promoter methylation reduced the luciferase activities by over 30-40-fold in Huh1 and Huh7 cells (Figure 7C). These results indicate that the hypermethylation of *GLS2* promoter greatly reduces the transcriptional activities of the *GLS2* promoter.

Methytransferase inhibitor 5-Aza-2’-deoxycytidine (5-Aza-dC) is a widely used DNA-demethylating agent, which induces expression of many genes with hypermethylated promoters [26, 27]. To further investigate whether hypermethylation of *GLS2* promoter is an important mechanism leading to the down-regulation of *GLS2* expression in HCCs, multiple HCC cell lines, including Huh1, Huh7, Hep3B and PLC/PRF/5 cells, were treated with 5-Aza-dC. MSP analysis showed that 5-Aza-dC greatly reduced the methylation of CpG sites in the *GLS2* promoter in these 4 HCC cell lines (Figure 7D). Notably, 5-Aza-dC greatly induced the mRNA levels of *GLS2* in these 4 HCC cell lines (by 3-7 folds) as measured by Taqman real-time PCR assays (Figure 7E), indicating that demethylation of the *GLS2* promoter increased GLS2 expression in HCC cells. Taken together, results in Figures 6 &7 suggest that GLS2 promoter hypermethylation is an important mechanism contributing to the decreased expression of GLS2 in HCCs.

### Loss of heterozygosity of GLS2 in primary HCCs

Loss of heterozygosity (LOH) is frequently observed in various tumors, including HCC, which leads to the decreased expression and loss of function of many tumor suppressor genes [28, 29]. To determine whether LOH contributes to the decreased expression of GLS2 in HCC, Taqman real-time PCR copy number assays were employed to determine the copy number of the gene in aforementioned 21 primary HCCs and their adjacent non-tumor liver tissues. Three different copy number assays, which measured the 5’, 3’ and the central region of the *GLS2* gene, respectively, were employed and the same results were obtained. Only 1 HCC lost a copy of GLS2 gene whereas the rest 20 HCCs retained both copies of *GLS2* (Table 1), indicating that although LOH occurs in *GLS2* gene in HCC, LOH is not a major mechanism accounting for the frequently observed down-regulation of GLS2 in HCC.

**Table 1:** LOH of *GLS2* in human primary HCCs

Copy number of GLS2	Non-Tumor	Tumor
1	0	1
2	21	20
Total	21	21

LOH of *GLS2* in HCCs was detected by Taqman copy number assays. Three different copy number assays, which measured the 5’, 3’ and the central region of the *GLS2* gene, respectively, were employed and the same results were obtained. Primary HCCs and their matched adjacent non-tumor liver samples (n=21; MD Anderson Cancer Center) were used for assays.

## DISCUSSION

GLS2 was recently identified as a novel p53 target gene [11, 12]. However, the role of GLS2 in tumorigenesis is unclear. In this study, we found that the GLS2 protein expression was significantly decreased in majority of primary HCCs that we examined compared with non-tumor liver tissues, including liver tissues with cirrhosis and hepatitis (Supplementary Table S2). This result is consistent with previous reports from our group and another group showing that GLS2 mRNA expression was greatly decreased in majority of HCCs [11, 12]. These results clearly demonstrated that the down-regulation of GLS2 is a common event in primary HCCs, which has the potential to be developed as a biomarker for early detection and diagnosis of HCCs. Our results further showed that ectopic GLS2 expression in HCC cells greatly reduced the anchorage-independent growth of cells and the growth of xenograft tumors. Consistently, knockdown of GLS2 in HCC cells clearly promoted the anchorage-independent growth of cells and growth of xenograft tumors. Our results demonstrated an important role of GLS2 in tumor suppression in HCC. In addition to the liver, GLS2 is also specifically expressed in the brain tissues. Interestingly, it was reported that the expression of GLS2 is also reduced in brain tumors [30]. It has been well-established that p53 plays a critical role in prevention of HCC [4, 5]. As a direct p53 target, GLS2 could contribute greatly to the function of p53 in tumor suppression in HCC.

The aberrant activation of the PI3K/AKT signaling is frequently observed in HCC, which plays a critical role in liver tumorigenesis [16-18]. Results from this study showed that GLS2 is an important negative regulator of the PI3K/AKT signaling in HCC; ectopic GLS2 expression clearly reduced AKT activities in HCC cells, whereas GLS2 knockdown enhanced AKT activities. Blocking the PI3K/AKT signaling in HCC cells largely abolished the inhibitory effect of GLS2 on the anchorage-independent cell growth and xenograft tumor growth. These results strongly suggest that the negative regulation of PI3K/AKT signaling contributes greatly to GLS2’s role in suppression of HCC. It remains unclear how GLS2 negatively regulates the PI3K/AKT signaling. GLS2 was reported to interact with other proteins through the PDZ domain in its C-terminus [31], which could contribute to its function in negative regulation of the PI3K/AKT signaling. Recently, GLS2 was also reported to regulate the expression of genes, although its mechanism is unclear [30]. Therefore, it is possible that GLS2 may change the expression of genes in the PI3K/AKT signaling pathway, contributing to the down-regulation of the PI3K/AKT signaling in cells. Further studies should shed light on the underlying mechanisms.

Our results further showed that the promoter region of *GLS2* is hypermethylated in a high percentage of HCCs but not in their matched adjacent non-tumor liver tissues. The hypermethylation of *GLS2* promoter was also observed in different HCC cell lines. Demethylation of *GLS2* promoter by 5-Aza-dC greatly induced GLS2 expression in these cells. Luciferase reporter assays further showed that in vitro methylation of CpG sites in *GLS2* promoter region greatly reduced the transcriptional activities of the *GLS2* promoter. These results strongly suggest that *GLS2* promoter hypermethylation is an important mechanism contributing to the decreased GLS2 expression in HCC.

In summary, the results in this study clearly demonstrated that GLS2 protein expression is significantly decreased in majority of HCC, and hypermethylation of GLS2 promoter is an important mechanism that contributes to the decreased expression of GLS2 in HCC. Our results further showed that GLS2 plays an important role in tumor suppression in HCC, and the negative regulation of the PI3K/AKT signaling by GLS2 contributes greatly to the function of GLS2 in tumor suppression in HCC.

## MATERIALS AND METHODS

### Cell lines, vectors, and cell treatments

Huh-1, Huh7, PLC/PRF/5, and Hep3B are human HCC cell lines. The pLPCX-GLS2 retroviral vector which expresses GLS2 was constructed by PCR amplification as described [11]. The pLHCX-DN-AKT retroviral vector expressing a dominant negative AKT (DN-AKT; K179M) was constructed by subcloning the DN-AKT DNA fragment from pLNCX-AKT1 K179M (Addgene) into the pLHCX vectors [22]. Two lentiviral shRNA vectors against *GLS2* (ID: V3LHS-307701; V2LHS-71048) and control shRNA vectors were obtained from Open Biosystems (Huntsville, AL). To establish cell lines with stable GLS2 knockdown, cells infected with shRNA vectors were selected by puromycin. For 5-Aza-2’-deoxycytidine (5-Aza-dC) treatments, cells were treated with 5 μM 5-AZA-dC (Sigma) for 7 days and 5-AZA-dC was replaced every day. Control cells were treated with DMSO.

### Tissue samples

Three tissue microarrays (TMAs) which include totally 110 HCC samples and 125 non-tumor liver tissues was provided from US Biomax (Rockville, MD). Twenty-one cases of formalin-fixed and paraffin-embedded (FFPE) primary HCC samples and their matched adjacent non-tumor liver tissues were collected at the University of Texas MD Anderson Cancer Center with approved IRB. Three fresh frozen non-tumor liver samples were provided by Origene (Rockville, MD).

### Immunohistochemistry (IHC) assays

The expression levels of GLS2 in tissues were determined by IHC assays as previously described [32]. GLS2 antibody was prepared as previously described [11]. Tyramide Signal Amplification plus dinitrphenyl (DNP)-HRP system (Perkin Elmer) was employed to increase the IHC signal. Rabbit IgG, replacing the primary antibody, was used as a negative control. Two known normal liver tissues were used as positive controls to normalize the staining efficiency from batch to batch. The IHC results were scored according to the percentage of tumor cells showing positive GLS2 staining: -: 0%; ±: < 10%; +: ≥10%.

### Taqman real-time PCR and semi-quantitative RT-PCR assays

Total RNA from cells and fresh frozen tissues was prepared by using an RNeasy kit (Qiagen). Total RNA from FFPE tissues was prepared by using an RNeasy FFPE kit (Qiagen). cDNA was prepared using a TaqMan reverse transcription kit. Taqman real-time PCR was performed using TaqMan PCR mixture (Applied Biosystems) as previously described [11]. The expression levels of the genes were normalized with *Actin* gene. The primers used for semi-quantitative RT-PCR were as follows: For *GLS2*, 5’-AGGCGAGAGTGTGCTGAGTGCTG-3’ and 5’-GCTGGTCCCCCTATGGCTGTTC-3’; For *Actin*, 5’-GGACTTCGAGCA AGAGATGG-3’ and 5’- AGCACTGTGTTGGCGTACAG-3’.

### Bisulfite treatment and promoter CpG methylation analysis

Genomic DNA from cells and fresh frozen tissues was extracted by using a QIAamp DNA mini Kit (Qiagen). Genomic DNA from FFPE tissues was extracted by using a QIAamp DNA FFPE Kits (Qiagen). DNA was treated with bisulfite to convert unmethylated cytosines into uracil while methylated cytosines remain unchanged by using EZ DNA Methylation Kit (Zymo Research). Methylation-specific PCR (MSP) and bisulfite genomic sequencing (BGS) were employed for *GLS2* promoter methylation analysis, respectively [33, 34]. For MSP, bisulfite-treated DNA was PCR amplified with either methylation-specific or unmethylation-specific primers. Sequences of methylation-specific primers: 5’-CGTTATTCGT CGGGTTTTGGGC-3’ (forward; at nt −398 to −377 relative to transcription start site) and 5’- GAAAACACCGTAAAACTACGAATAATAAAATTATCG-3’ (reverse; at nt −275 to −240); Sequences of unmethylation-specific primers: 5’- TTATGTTATTTGTTGGGTTTTGGGTGTTG-3’ (forward; at nt −401 to −373) and 5’- CCAAAAACACCATAAAACTACAAATAATAAAATTATCA-3’ (reverse; at nt −275 to −238). For BGS analysis, the bisulfite-treated DNA was used to amplify the promoter region of *GLS2* (nt −460 to +86 relative to the transcription start site) and cloned into TA vectors (Invitrogen). Eight different clones were sequenced to examine the CpG methylation in *GLS2* promoter.

### Construction of luciferase reporter vectors, in vitro methylation and luciferase activity assays

The *GLS2* promoter region (-458 to +86 relative to the transcription start site) was PCR amplified and subcloned into pGL2 luciferase reporter vectors (Promega) at BglII and HindIII sites. *In vitro* methylation of *GLS2* promoter was performed as described [24, 25]. In brief, the luciferase reporter vectors containing *GLS2* promoter (10 μg) were methylated using CpG methyltransferase M.SssI, which methylates all cytosine residues of CpG. The completion of CpG methylation was confirmed by digestion of HpaII, a methylation-sensitive restriction enzyme which can not cut CCGG sites when CpG is methylated. In a parallel control reaction, the reporter vectors containing *GLS2* promoter (10 μg) were mock-methylated. Methylated and mock-methylated reporter vectors were then digested with BglII and HindIII, and the *GLS2* promoter DNA fragments were purified and then re-ligated into unmethylated pGL2 vectors, which were digested with BglII and HindIII. After ligation, the unmethylated pGL2 vectors containing methylated or mock-methylated *GLS2* promoter were used directly for luciferase reporter assays. Luciferase reporter assays were performed as described [35]. Briefly, the reporter vectors containing methylated or mock-methylated *GLS2* promoter were transfected into cells along with pRL-SV40 vectors expressing *renilla* luciferase as an internal control to normalize the transfection efficiency. The luciferase activity was measured 24 h after transfection.

### Taqman real-time gene copy number analysis

Gene copy number was determined by duplex Taqman copy number assays using Taqman genotype Mix [32, 36]. FAM-labeled primers for GLS2 and VIC-labeled primer for TERT as an internal control were obtained from Applied Biosystems. Three primers (Hs01976936_cn, Hs02840568_cn and Hs03075375_cn) which measure the 5’ (intron 2-exon 3), 3’ (intron 15-exon 16) and the central region of the *GLS2* gene (intron 10-exon 11), respectively, were employed. The ΔΔCt method was used for data analysis.

### Western-blot Analysis

Western-blot assays were performed as previously described [11]. Following antibodies were used. Anti-p-AKT (Ser473) (#4051, Cell signaling), Anti-p-AKT (Thr308) (#4056, Cell signaling), Anti-AKT (Sc-1618, Santa Cruz). Anti-Flag-M2 (F1804, Sigma), Anti-*β*-Actin (A5441, Sigma). GLS2 antibody was prepared as previously described [11]. The protein levels were quantified by digitalization of the X-ray film and analyzed with Scion Image software (Scion Corporation, Frederick, MD).

### Anchorage-independent growth assays

Anchorage-independent growth assays were performed in dishes coated with media containing 0.6% agarose. Cells were seeded on top of this layer in media containing 0.3% agarose. Colonies were stained and counted after 2-3 weeks [37].

### Xenograft tumorigencity analysis

Xenograft tumorigenicity assays were performed as previously described [38]. Seven-week-old BALB/c nu/nu male athymic nude mice (Taconic) were used. Cells (6x 10^6^ for Huh1 cells, and 1x 10^7^ for Huh7 and PLC/PRF/5 in 0.2 mL PBS) were injected (s.c.) into nude mice (n = 10/group). After injection, mice were examined and tumor volumes were measured 3 times/week for 3-5 weeks. Tumor volume = ½ (length × width^2^). All experiments with animals were performed with the approval of the Institutional Animal Care and Use Committee.

### Statistical analysis

The differences in tumor growth among groups were analyzed for statistical significance by ANOVA, followed by Student’s *t*-tests. All other *P* values were obtained using Student *t*-tests or χ2 tests. **: *p*<0.001; *: *p*<0.01; #: *p*<0.05.

## SUPPLEMENTARY FIGURES AND TABLES



## References

[1] Levine AJ, Hu W, Feng Z (2006). The P53 pathway: what questions remain to be explored?. Cell Death Differ.

[2] Vousden KH, Prives C (2009). Blinded by the Light: The Growing Complexity of p53. Cell.

[3] Levine AJ, Oren M (2009). The first 30 years of p53: growing ever more complex. Nat Rev Cancer.

[4] Greenblatt MS, Bennett WP, Hollstein M, Harris CC (1994). Mutations in the p53 tumor suppressor gene: clues to cancer etiology and molecular pathogenesis. Cancer Res.

[5] Staib F, Hussain SP, Hofseth LJ, Wang XW, Harris CC (2003). TP53 and liver carcinogenesis. Hum Mutat.

[6] Donehower LA, Harvey M, Slagle BL, McArthur MJ, Montgomery CA, Butel JS, Bradley A (1992). Mice deficient for p53 are developmentally normal but susceptible to spontaneous tumours. Nature.

[7] Jacks T, Remington L, Williams BO, Schmitt EM, Halachmi S, Bronson RT, Weinberg RA (1994). Tumor spectrum analysis in p53-mutant mice. Curr Biol.

[8] Feng Z, Levine AJ (2010). The regulation of energy metabolism and the IGF-1/mTOR pathways by the p53 protein. Trends Cell Biol.

[9] Perez-Gomez C, Mates JM, Gomez-Fabre PM, del Castillo-Olivares A, Alonso FJ, Marquez J (2003). Genomic organization and transcriptional analysis of the human l-glutaminase gene. Biochem J.

[10] Campos-Sandoval JA, Lopez de la Oliva AR, Lobo C, Segura JA, Mates JM, Alonso FJ, Marquez J (2007). Expression of functional human glutaminase in baculovirus system: affinity purification, kinetic and molecular characterization. Int J Biochem Cell Biol.

[11] Hu W, Zhang C, Wu R, Sun Y, Levine A, Feng Z (2010). Glutaminase 2, a novel p53 target gene regulating energy metabolism and antioxidant function. Proc Natl Acad Sci U S A.

[12] Suzuki S, Tanaka T, Poyurovsky MV, Nagano H, Mayama T, Ohkubo S, Lokshin M, Hosokawa H, Nakayama T, Suzuki Y, Sugano S, Sato E, Nagao T, Yokote K, Tatsuno I, Prives C (2010). Phosphate-activated glutaminase (GLS2), a p53-inducible regulator of glutamine metabolism and reactive oxygen species. Proc Natl Acad Sci U S A.

[13] Cancer Facts & Figures 2013 (2013).

[14] Forner A, Llovet JM, Bruix J (2012). Hepatocellular carcinoma. Lancet.

[15] Jemal A, Bray F, Center MM, Ferlay J, Ward E, Forman D (2011). Global cancer statistics. CA: a cancer journal for clinicians.

[16] Engelman JA (2009). Targeting PI3K signalling in cancer: opportunities, challenges and limitations. Nat Rev Cancer.

[17] Carnero A, Blanco-Aparicio C, Renner O, Link W, Leal JF (2008). The PTEN/PI3K/AKT signalling pathway in cancer, therapeutic implications. Curr Cancer Drug Targets.

[18] Vivanco I, Sawyers CL (2002). The phosphatidylinositol 3-Kinase AKT pathway in human cancer. Nat Rev Cancer.

[19] Esteller M (2007). Cancer epigenomics: DNA methylomes and histone-modification maps. Nat Rev Genet.

[20] Herath NI, Leggett BA, MacDonald GA (2006). Review of genetic and epigenetic alterations in hepatocarcinogenesis. Journal of gastroenterology and hepatology.

[21] Baylin SB, Esteller M, Rountree MR, Bachman KE, Schuebel K, Herman JG (2001). Aberrant patterns of DNA methylation, chromatin formation and gene expression in cancer. Hum Mol Genet.

[22] Ramaswamy S, Nakamura N, Vazquez F, Batt DB, Perera S, Roberts TM, Sellers WR (1999). Regulation of G1 progression by the PTEN tumor suppressor protein is linked to inhibition of the phosphatidylinositol 3-kinase/Akt pathway. Proc Natl Acad Sci U S A.

[23] Aoki M, Batista O, Bellacosa A, Tsichlis P, Vogt PK (1998). The akt kinase: molecular determinants of oncogenicity. Proc Natl Acad Sci U S A.

[24] Dell G, Charalambous M, Ward A (2001). In vitro methylation of specific regions in recombinant DNA constructs by excision and religation. Methods Mol Biol.

[25] To KK, Zhan Z, Bates SE (2006). Aberrant promoter methylation of the ABCG2 gene in renal carcinoma. Mol Cell Biol.

[26] Michalowsky LA, Jones PA (1987). Differential nuclear protein binding to 5-azacytosine-containing DNA as a potential mechanism for 5-aza-2'-deoxycytidine resistance. Mol Cell Biol.

[27] Soengas MS, Capodieci P, Polsky D, Mora J, Esteller M, Opitz-Araya X, McCombie R, Herman JG, Gerald WL, Lazebnik YA, Cordon-Cardo C, Lowe SW (2001). Inactivation of the apoptosis effector Apaf-1 in malignant melanoma. Nature.

[28] Kondoh N, Wakatsuki T, Hada A, Shuda M, Tanaka K, Arai M, Yamamoto M (2001). Genetic and epigenetic events in human hepatocarcinogenesis. International journal of oncology.

[29] Midorikawa Y, Tang W, Sugiyama Y (2007). High-resolution mapping of copy number aberrations and identification of target genes in hepatocellular carcinoma. Bioscience trends.

[30] Szeliga M, Obara-Michlewska M, Matyja E, Lazarczyk M, Lobo C, Hilgier W, Alonso FJ, Marquez J, Albrecht J (2009). Transfection with liver-type glutaminase cDNA alters gene expression and reduces survival, migration and proliferation of T98G glioma cells. Glia.

[31] Olalla L, Gutierrez A, Jimenez AJ, Lopez-Tellez JF, Khan ZU, Perez J, Alonso FJ, de la Rosa V, Campos-Sandoval JA, Segura JA, Aledo JC, Marquez J (2008). Expression of the scaffolding PDZ protein glutaminase-interacting protein in mammalian brain. J Neurosci Res.

[32] Hu W, Feng Z, Modica I, Klimstra DS, Song L, Allen PJ, Brennan MF, Levine AJ, Tang LH (2010). Gene Amplifications in Well-Differentiated Pancreatic Neuroendocrine Tumors Inactivate the p53 Pathway. Genes Cancer.

[33] Licchesi JD, Herman JG (2009). Methylation-specific PCR. Methods Mol Biol.

[34] Li Y, Tollefsbol TO (2011). DNA methylation detection: bisulfite genomic sequencing analysis. Methods Mol Biol.

[35] Hu W, Feng Z, Teresky AK, Levine AJ (2007). p53 regulates maternal reproduction through LIF. Nature.

[36] Mayo P, Hartshorne T, Li K, McMunn-Gibson C, Spencer K, Schnetz-Boutaud N, Jonathan L Haines (2010). CNV analysis using TaqMan copy number assays. Current protocols in human genetics / editorial board.

[37] Zhang C, Liu J, Liang Y, Wu R, Zhao Y, Hong X, Lin M, Yu H, Liu L, Levine AJ, Hu W, Feng Z (2013). Tumour-associated mutant p53 drives the Warburg effect. Nature communications.

[38] Hu W, Chan CS, Wu R, Zhang C, Sun Y, Song JS, Tang LH, Levine AJ, Feng Z (2010). Negative regulation of tumor suppressor p53 by microRNA miR-504. Molecular cell.

