# Supplementary material for: Glutaminase 2 negatively regulates the PI3K/AKT signaling and shows tumor suppression activity in human hepatocellular carcinoma
Source: Oncotarget. 2014 Mar 26;5(9):2635–47. doi: 10.18632/oncotarget.1862 (PMC4058033; doi:10.18632/oncotarget.1862)

Supplementary Information

Supplementary Table S1. The GLS2 protein expression in 110 HCC specimens

Clinico-pathological characteristic	Categorisation	n (%)	GLS2 expression			*p*
			-	±	+	
Age						
	<55 years	70 (63.6)	61	5	4	>0.05
	≥55 years	40 (36.4)	35	5	0	
Gender						
	Female	26 (23.6)	25	0	1	>0.05
	male	84 (76.4)	71	10	3	
Tumor stage						
	I	1 (0.9)	1	0	0	>0.05
	II	50 (45.5)	45	4	1	
	III	59 (53.6)	50	6	3	
Histological grade						
	1	6 (5.4)	5	0	1	>0.05
	2	65 (59.1)	57	7	1	
	3	31 (28.2)	27	2	2	
	NA	8 (7.3)	7	1	0	

The GLS2 protein expression in 110 HCC specimens in three TMAs (US Biomax) was determined by IHC assays. -: 0% positive staining cells; ±: <10% positive staining cells; +: ≥10% positive staining cells. The *p* values were calculated using $\chi 2$ tests. NA: Information not available.

Supplementary Table S2. The GLS2 protein expression in 125 non-tumor specimens

Clinico-pathological characteristic	Categorisation	n (%)	GLS2 expression			*p*
			-	±	+	
Age						
	<55 years	88 (70.4)	0	5	83	>0.05
	≥55 years	37 (29.6)	0	2	35	
Gender						
	Female	31 (24.8)	0	2	29	>0.05
	Male	94 (75.2)	0	5	89	
Pathology						
	Cirrhosis	43 (34.4)	0	2	41	>0.05
	Hepatitis	12 (9.6)	0	1	11	
	Normal	70 (56)	0	4	66	

The GLS2 protein expression in 125 non-tumor specimens in three TMA (US Biomax) was determined by IHC assays. -: 0% positive staining cells; ±: <10% positive staining cells; +: ≥10% positive staining cells. The *p* values were calculated using $\chi 2$ tests.

Supplementary Table S3. The GLS2 protein expression in 21 HCC specimens

Clinico-pathological characteristic	Categorisation	n (%)	GLS2 expression			*p*
			-	±	+	
Age						
	<55 years	5 (24)	4	0	1	>0.05
	≥55 years	16 (76)	15	1	0	
Gender						
	Female	6 (28)	5	0	0	>0.05
	male	15 (72)	14	1	1	
Tumor stage						
	I	5 (24)	4	0	1	>0.05
	II	12 (57)	11	1	0	
	III	4 (19)	4	0	0	
Histological grade						
	1	1 (5)	1	0	0	>0.05
	2	10 (47)	9	0	1	
	3	8 (38)	7	1	0	
	4	2 (9)	2	0	0	

The GLS2 protein expression in 21 HCC specimens (MD Anderson Cancer Center) was determined by IHC assays. -: 0% positive staining cells; ±: <10% positive staining cells; +: ≥10% positive staining cells. The *p* values were calculated using $\chi 2$ tests.

Supplementary Figure Legend:

Supplementary Figure S1: The down-regulation of GLS2 expression in human primary HCCs. Twenty-one pairs of primary HCCs and their matched adjacent non-tumor liver tissues were collected at MD Anderson Cancer Center. (A) The GLS2 expression in HCCs measured by IHC assays. Left panel: Representative IHC staining of GLS2 in 2 HCCs which showed negative staining (-) and matched non-tumor liver tissues, which showed positive staining (+). Right panel: IHC staining results of 21 HCCs and their adjacent non-tumor tissues. -: 0% positive staining cells; ±: <10% positive staining cells; +: >10% positive staining cells. The *p* values were calculated using $\chi 2$ tests. (B) The GLS2 mRNA levels were measured by Taqman real-time PCR assays and normalized with Actin. The relative GLS2 mRNA levels in HCCs and their adjacent non-tumor liver tissues were calculated by comparing with the GLS2 mRNA levels in the adjacent liver tissue of HCC #1, which were designated as 1. Data are presented as mean ± SD (n = 3).

Supplementary Figure S1

A

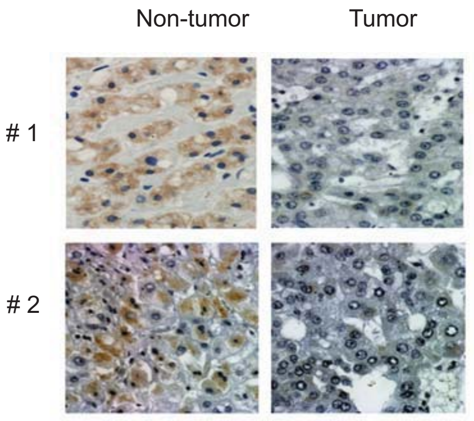

GLS2 staining	Non-tumor	Tumor
	n	n
-	0	19
±	1	1
+	20	1
Total	21	21

p<0.0001, χ^2 test

B

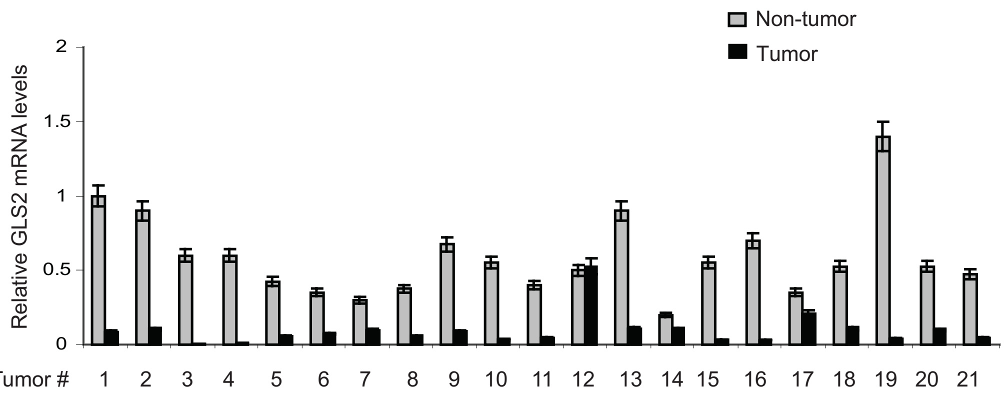

Supplement: Supplementary file 1 [file oncotarget-05-2635-s001.pdf]
